# Supplementary figures and images for: Convergent antibody evolution and clonotype expansion following influenza virus vaccination
Source: PLoS One. 2021 Feb 22;16(2):e0247253. doi: 10.1371/journal.pone.0247253 (PMC7899375; doi:10.1371/journal.pone.0247253)

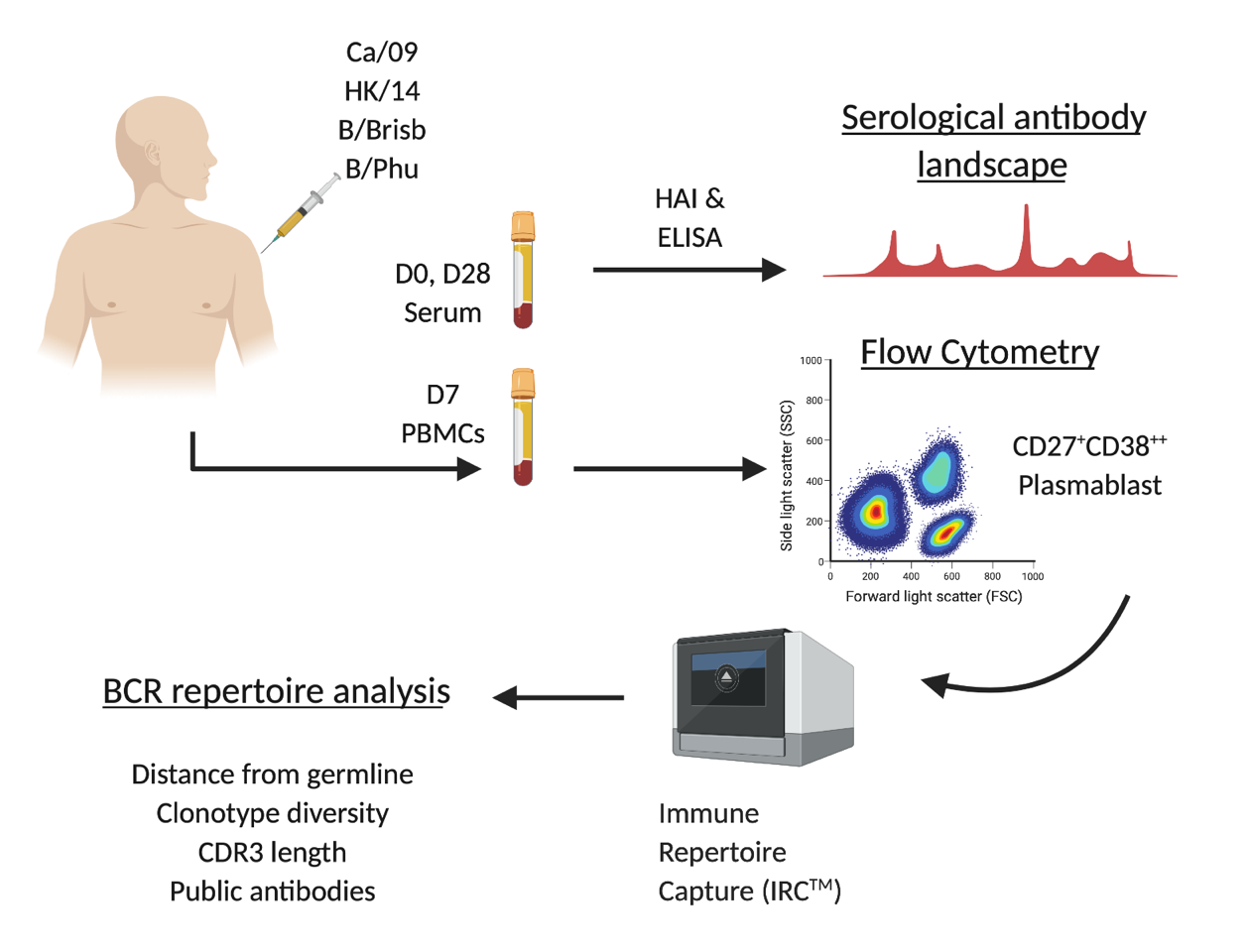

Supplement: S1 Graphical abstract — PBMCs were collected 7 days after influenza virus vaccination. Sorted PBs were subjected to the Immune Repertoire CaptureTM. Cells were barcoded to identify paired heavy and light chain sequences that were used in the downstream BCR repertoire analyses. (TIFF) [file pone.0247253.s001.tiff]

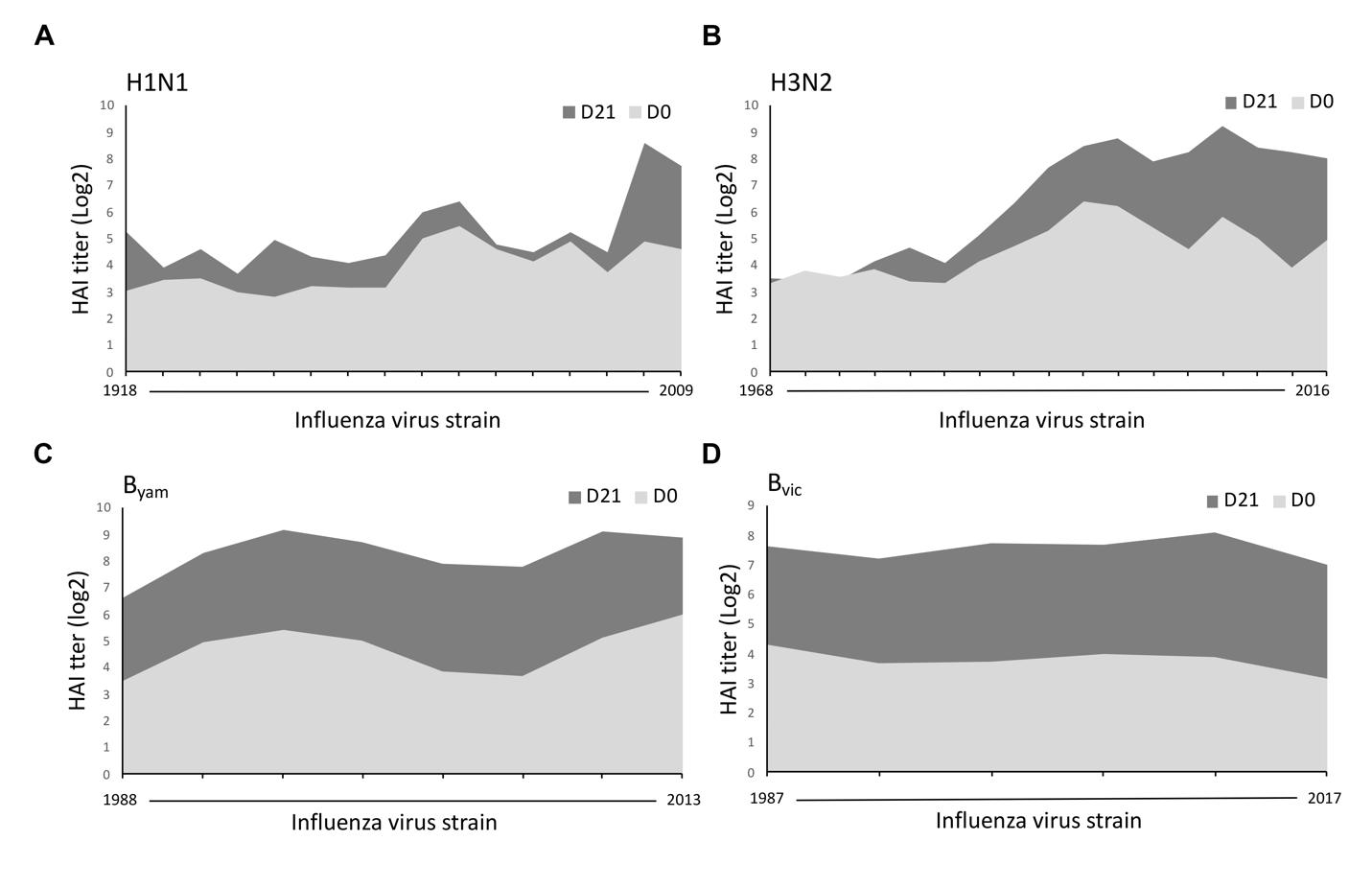

Supplement: S1 Fig — Serum samples from 17 subjects with significant serological response to influenza vaccine were tested for HAI activity against a broad panel of (A) H1N1 IAV strains from 1918 to 2009, (B) H3N2 IAV strains from 1968 to 2016, (C) IBV Yamagata lineage virus from 1988 to 2013, and (D) IBV Victoria lineage from 1987 to 2017. (TIFF) [file pone.0247253.s002.tiff]

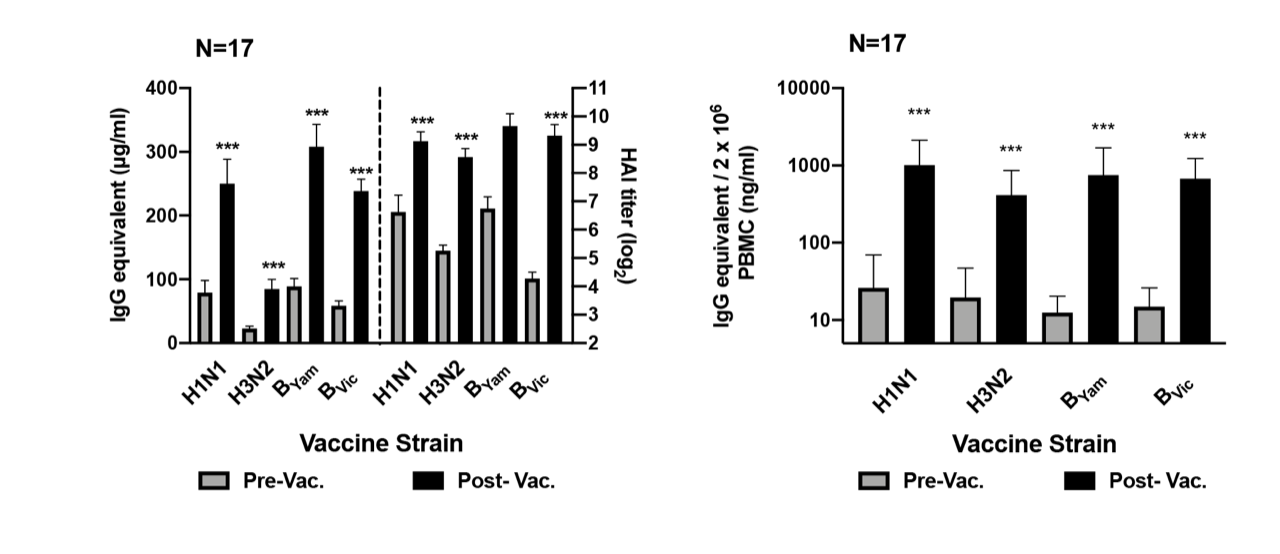

Supplement: S2 Fig — (A) Serological antibody responses to influenza vaccination in the 17 participants analyzed in this study. (B) Memory B cell-derived antibody responses to influenza vaccination in the 17 participants analyzed in this study. (TIFF) [file pone.0247253.s003.tiff]

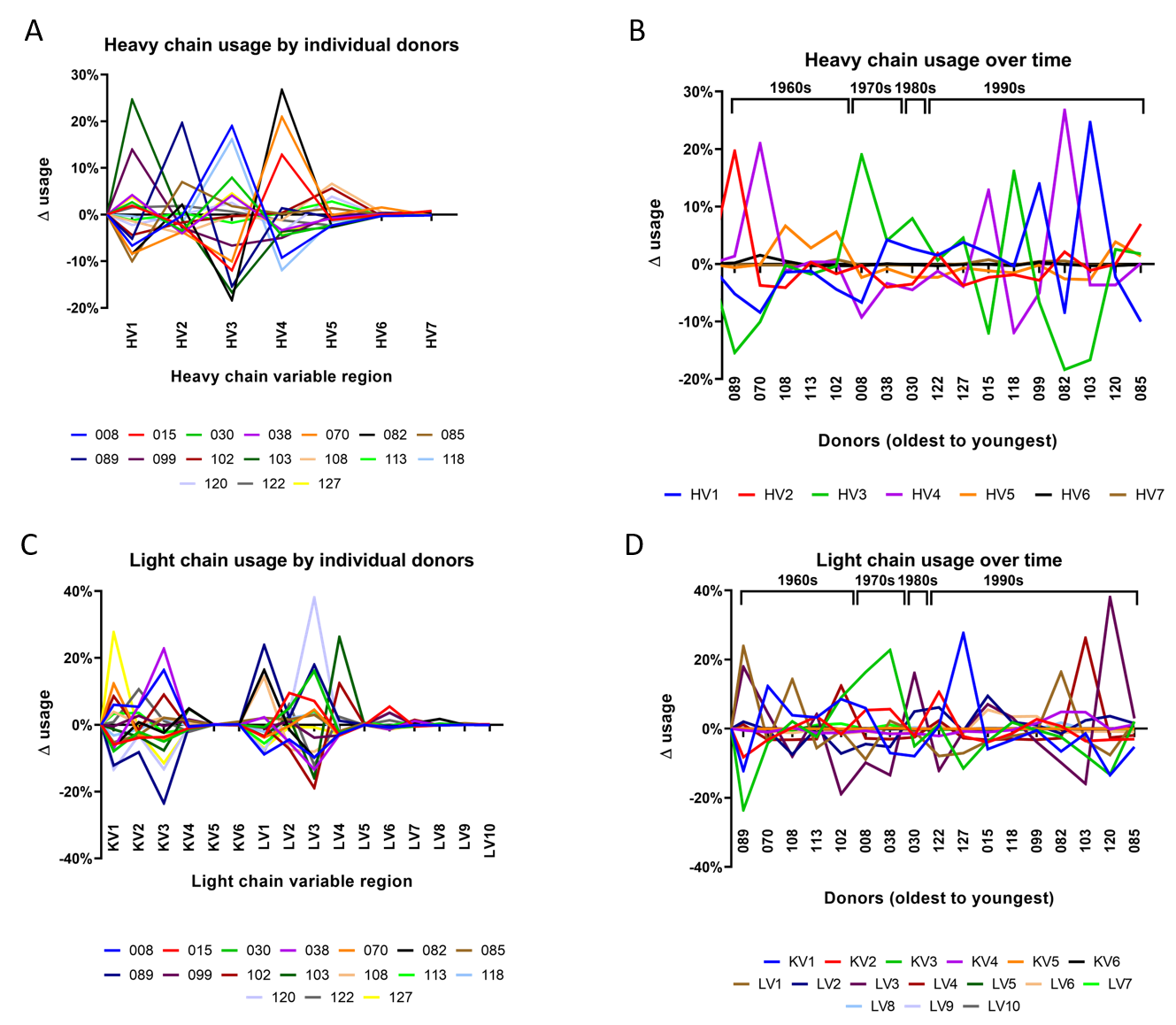

Supplement: S3 Fig — (A) Divergence from the mean heavy chain variable segment usage for each participant. (B) Divergence from the mean heavy chain variable segment usage by participant age. (C) Divergence from the mean light chain variable segment usage for each participant. (D) Divergence from the mean light chain variable segment usage by participant age. (TIFF) [file pone.0247253.s004.tiff]

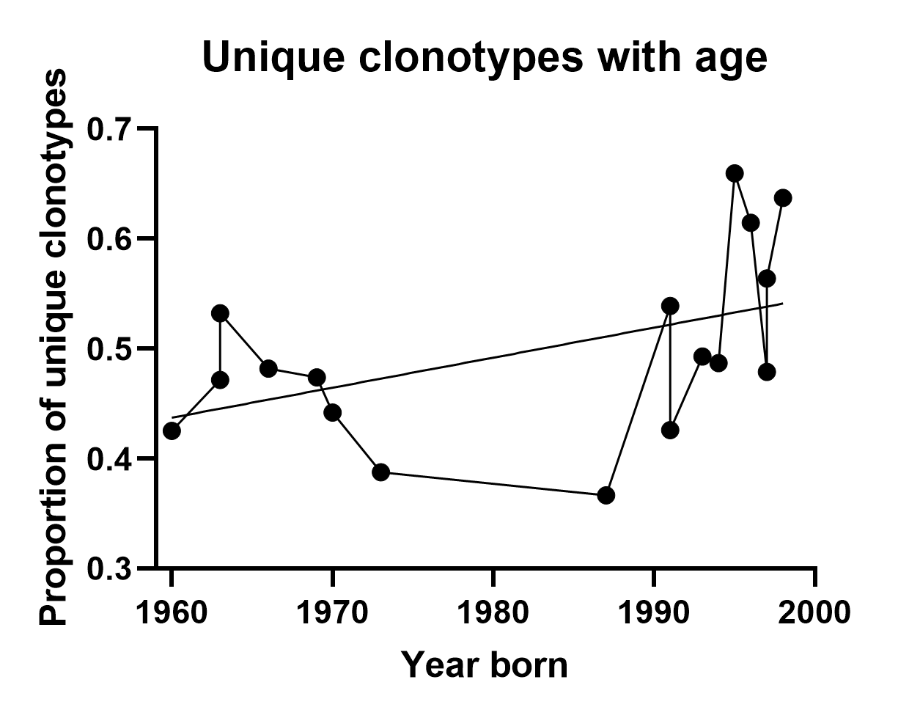

Supplement: S4 Fig — (TIFF) [file pone.0247253.s005.tiff]

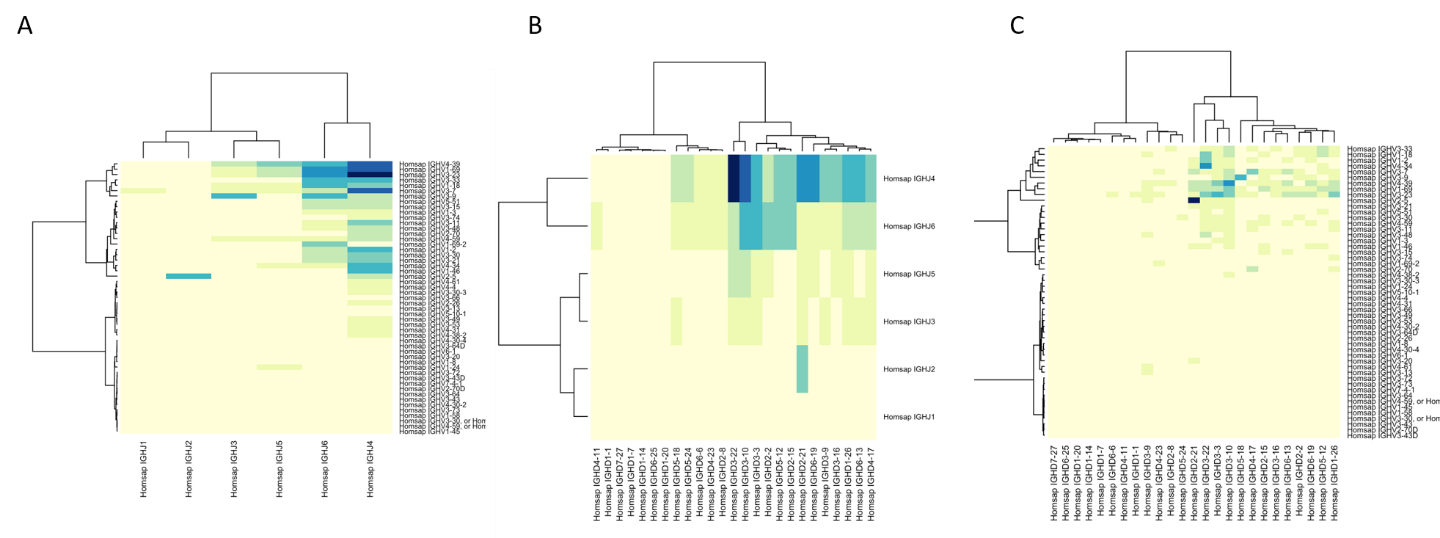

Supplement: S5 Fig — (A) Heatmap of preferential association between V and J gene segments. (B) Heatmap of preferential association between J and D gene segments. (C) Heatmap of preferential association between V and D gene segments. Yellow corresponds to low pairing frequency, while dark blue corresponds to the highest observed pairing frequency. (TIFF) [file pone.0247253.s006.tiff]

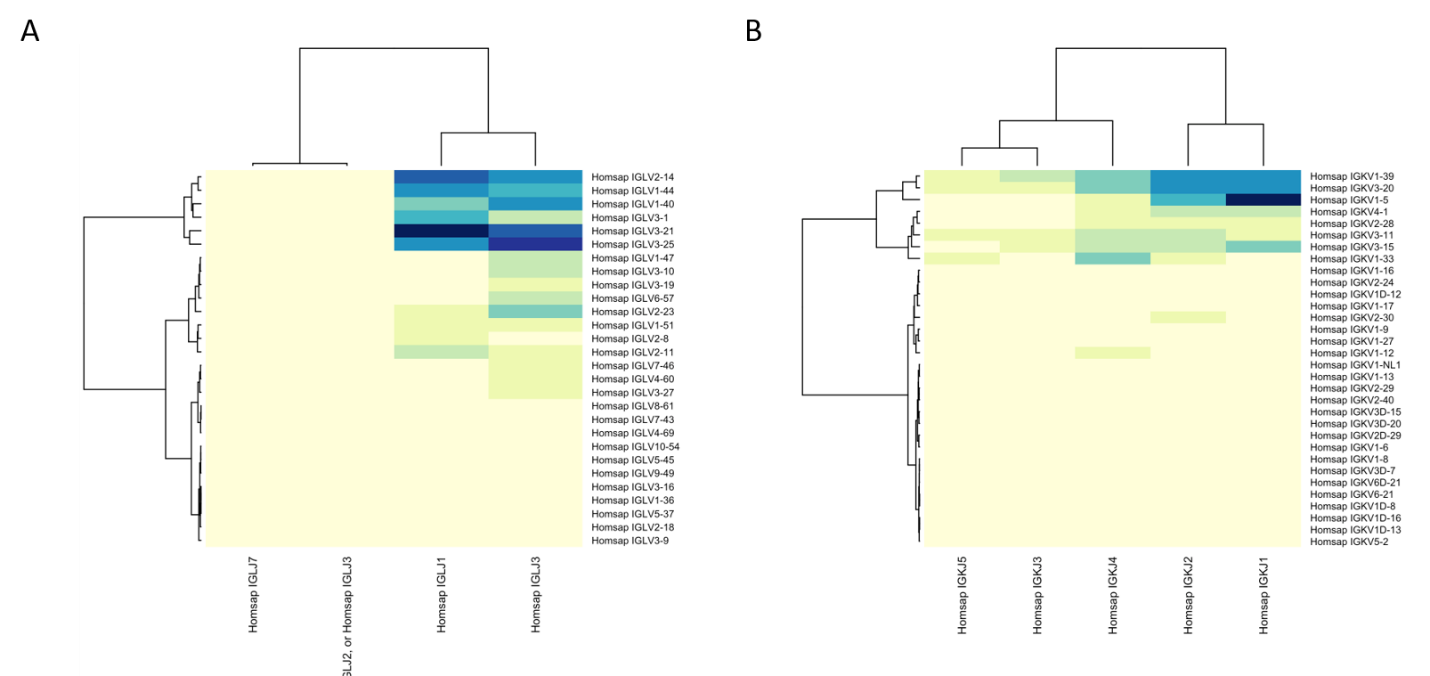

Supplement: S6 Fig — (A) Heatmap of preferential association between V and J gene segments for lambda light chains. (B) Heatmap of preferential association between V and J gene segments or kappa light chains. Yellow corresponds to low pairing frequency, while dark blue corresponds to the highest observed pairing frequency. (TIFF) [file pone.0247253.s007.tiff]

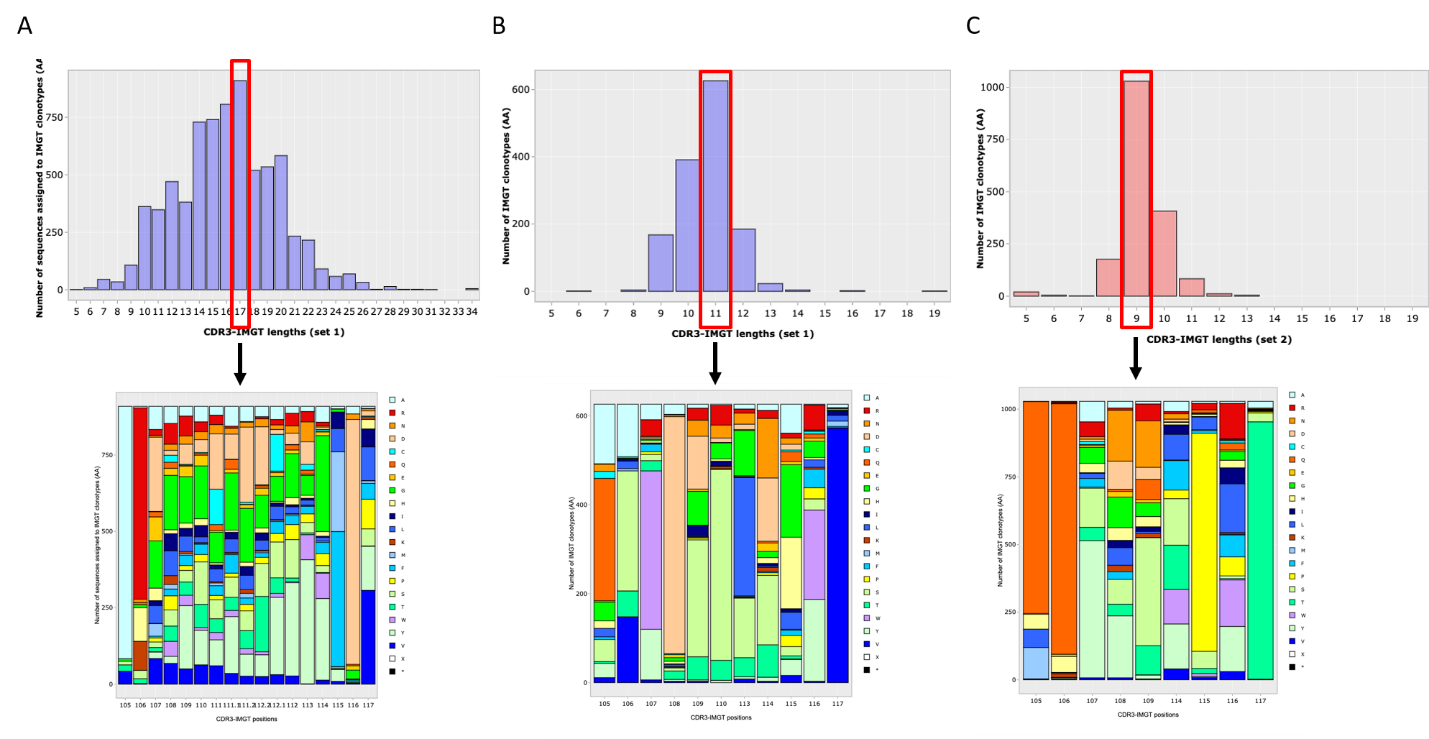

Supplement: S7 Fig — (A) HCDR3 length distribution and amino acid frequencies. (B) LCDR3 length distribution and amino acid frequencies for lambda light chains. (C) LCDR3 length distribution and amino acid frequencies for kappa light chains. (TIFF) [file pone.0247253.s008.tiff]

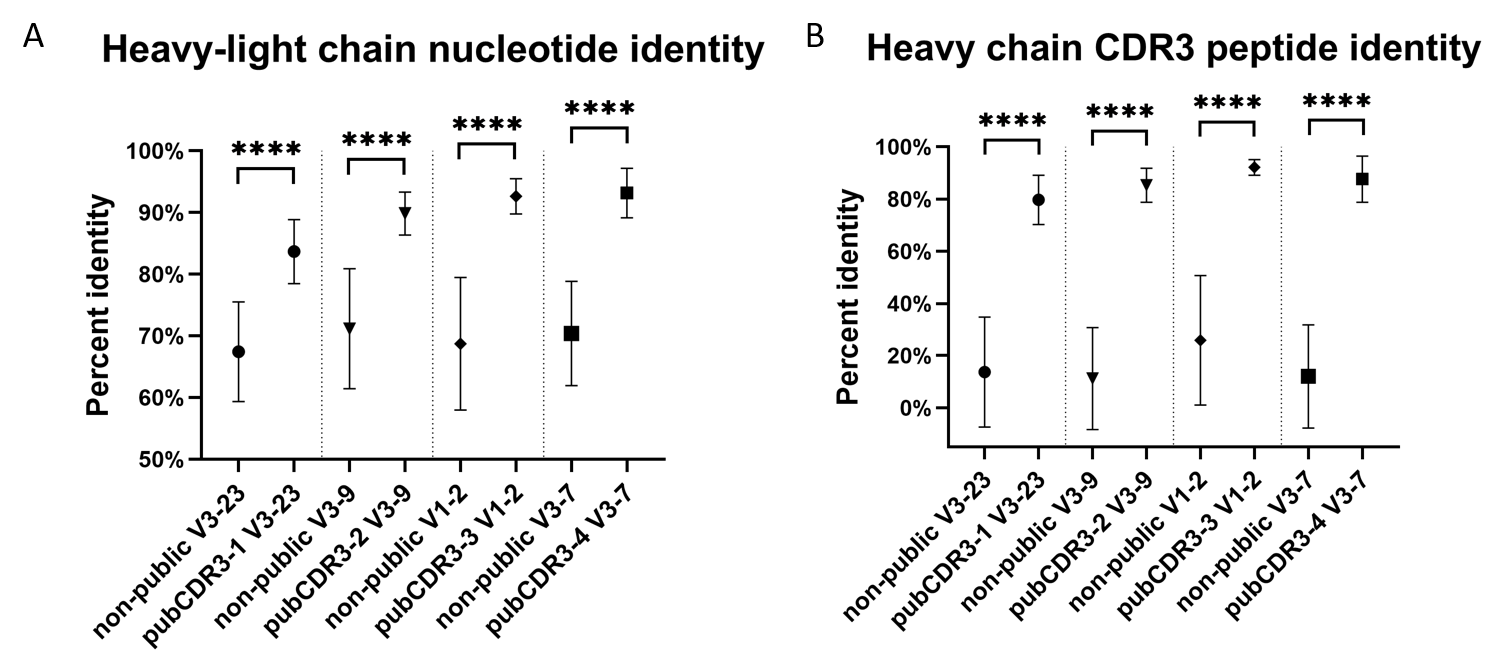

Supplement: S8 Fig — Public PBs are contrasted with non-public PBs belonging to the same heavy chain variable segment, based on (A) heavy chain variable segment nucleotide identity, and (B) HCDR3 peptide identity. (TIFF) [file pone.0247253.s009.tiff]
