# Supplementary material for: Convergent antibody evolution and clonotype expansion following influenza virus vaccination
Source: PLoS One. 2021 Feb 22;16(2):e0247253. doi: 10.1371/journal.pone.0247253 (PMC7899375; doi:10.1371/journal.pone.0247253)
Supplement: S1 Table — (DOCX) [file pone.0247253.s010.docx]

**S1 Table.** **PB response to influenza vaccination.**

| Participant ID# | DOB | Sex | # ASC/10^7^ PBMC | % Vac-specific ASC |
| --- | --- | --- | --- | --- |
| D#008 | 1970 | F | 33640 | 21.85 |
| D#015 | 1993 | F | 112000 | 1.55 |
| D#030 | 1987 | M | 35780 | 5.50 |
| D#038 | 1973 | M | 16640 | 23.60 |
| D#070 | 1963 | M | 22980 | 48.89 |
| D#082 | 1996 | F | 72110 | 5.75 |
| D#085 | 1998 | F | 87820 | 2.44 |
| D#089 | 1960 | M | 11680 | 47.80 |
| D#099 | 1995 | F | 132500 | 1.25 |
| D#102 | 1969 | M | 66110 | 2.74 |
| D#103 | 1997 | F | 54840 | 1.70 |
| D#108 | 1963 | M | 9810 | 16.33 |
| D#113 | 1966 | F | 13960 | 7.52 |
| D#118 | 1994 | F | 95420 | 4.09 |
| D#120 | 1997 | M | 41580 | 10.03 |
| D#122 | 1991 | F | 87240 | 8.45 |
| D#127 | 1991 | F | 77450 | 3.41 |
| Average |  |  | **57151** | **13** |
