# Supplementary material for: Convergent antibody evolution and clonotype expansion following influenza virus vaccination
Source: PLoS One. 2021 Feb 22;16(2):e0247253. doi: 10.1371/journal.pone.0247253 (PMC7899375; doi:10.1371/journal.pone.0247253)
Supplement: S2 Table — Colors correspond to those use in Fig 3. (DOCX) [file pone.0247253.s011.docx]

**S2 Table.** **List of the three most expanded clonotypes for each donor.** Colors correspond to those use in Fig 3.

| **Participant** | **Most expanded clonotypes** | **# of PBs in clonotype** |
| --- | --- | --- |
| D#008 | IGHV3-9/D6-13/J6/IGKV3-15/J4 | 101 |
|  | IGHV3-30/D3-10/J4/IGKV1-5/J1 | 60 |
|  | IGHV3-13/D3-9/J4/IGKV2-30/J5 | 19 |
| D#015 | IGHV4-34/D3-22/J4/IGKV1-39/J2 | 20 |
|  | IGHV1-69/D3-3/J6/IGLV3-1/J2 | 19 |
|  | IGHV4-38-2/D3-16/J4/IGLV2-8/J2 | 13 |
|  | IGHV4-39/D2-21/J5/IGLV3-21/J2 | 13 |
| D#030 | IGHV3-15/D2-15/J6/IGLV3-21/J2 | 60 |
|  | IGHV4-34/D2-21/J4/IGLV1-40/J1 | 33 |
|  | IGHV3-33/D3-16/J2/IGLV3-21/J2 | 27 |
| D#038 | IGHV3-33/D5-12/J6/IGKV3-11/J4 | 101 |
|  | IGHV3-30/D5-12/J6/IGKV3-11/J4 | 21 |
|  | IGHV4-38-2/D3-10/J4/IGLV1-47/J2 | 21 |
| D#070 | IGHV4-34/D3-22/J4/IGKV1-5/J2 | 32 |
|  | IGHV1-2/D4-17/J4/IGLV3-21/J2 | 25 |
|  | IGHV3-7//J4/IGLV1-44/J3 | 24 |
| D#082 | IGHV4-59/D2-15/J4/IGLV1-40/J2 | 15 |
|  | IGHV4-59/D5-24/J3/IGLV1-40/J2 | 12 |
|  | IGHV1-69/D3-10/J5/IGKV3-15/J1 | 11 |
| D#085 | IGHV3-9/D3-10/J4/IGLV2-23/J2 | 22 |
|  | IGHV2-70/D1-26/J4/IGLV3-21/J2 | 12 |
|  | IGHV3-48/D3-22/J6/IGKV3-20/J2 | 10 |
|  | IGHV3-23/D4-17/J6/IGLV3-21/J2 | 10 |
| D#089 | IGHV2-5/D2-21/J2/IGLV3-21/J1 | 104 |
|  | IGHV3-7/D4-23/J1/IGLV1-44/J1 | 19 |
|  | IGHV3-7/D4-23/J4/IGLV1-44/J1 | 12 |
| D#099 | IGHV1-69/D3-3/J5/IGLV6-57/J3 | 16 |
|  | IGHV3-66/D4-23/J2/IGLV3-21/J2 | 11 |
|  | IGHV3-21/D3-22/J4/IGLV3-21/J3 | 10 |
| D#102 | IGHV5-51/D2-15/J4/IGLV4-60/J3 | 31 |
|  | IGHV1-69/D3-16/J4/IGLV4-69/J2 | 23 |
|  | IGHV3-23/D1-26/J5/IGKV1-5/J1 | 21 |
| D#103 | IGHV1-69-2/D4-23/J6/IGLV4-60/J2 | 105 |
|  | IGHV3-15/D7-27/J4/IGKV4-1/J5 | 15 |
|  | IGHV1-69-2/D5-24/J6/IGLV4-60/J2 | 12 |
|  | IGHV1-69-2/D3-16/J6/IGLV4-60/J2 | 12 |
| D#108 | IGHV3-21/D6-13/J4/IGLV1-40/J1 | 23 |
|  | IGHV5-51/D6-19/J3/IGLV1-51/J2 | 16 |
|  | IGHV3-7/D4-17/J6/IGLV1-40/J2 | 14 |
|  | IGHV5-51/D2-21/J6/IGLV1-40/J3 | 14 |
| D#113 | IGHV3-7/D4-17/J6/IGLV3-21/J1 | 38 |
|  | IGHV3-30/D2-2/J6/IGKV1-5/J1 | 26 |
|  | IGHV3-15/D6-13/J3/IGKV1-33/J4 | 23 |
| D#118 | IGHV3-7/D6-13/J6/IGLV3-21/J2 | 40 |
|  | IGHV1-18/D5-12/J6/IGLV6-57/J2 | 24 |
|  | IGHV1-18/D5-12/J6/IGKV3-20/J2 | 19 |
| D#120 | IGHV3-30/D3-10/J4/IGLV2-8/J3 | 28 |
|  | IGHV3-23/D6-19/J4/IGLV1-47/J1 | 15 |
|  | IGHV2-5/D3-9/J3/IGKV4-1/J1 | 11 |
| D#122 | IGHV3-20/D2-21/J4/IGKV3-20/J3 | 13 |
|  | IGHV1-69/D5-12/J4/IGKV2-28/J1 | 12 |
|  | IGHV1-69/D6-19/J4/IGKV2-28/J1 | 11 |
| D#127 | IGHV3-9/D5-18/J6/IGKV1-33/J4 | 71 |
|  | IGHV3-9/D5-18/J3/IGKV1-5/J1 | 48 |
|  | IGHV3-9/D5-24/J6/IGKV1-33/J4 | 24 |
