# Supplementary material for: Convergent antibody evolution and clonotype expansion following influenza virus vaccination
Source: PLoS One. 2021 Feb 22;16(2):e0247253. doi: 10.1371/journal.pone.0247253 (PMC7899375; doi:10.1371/journal.pone.0247253)
Supplement: S3 Table — Colors correspond to those use in Fig 5. (DOCX) [file pone.0247253.s012.docx]

**S3 Table.** **List of convergent (public) clonotypes for each donor.** Colors correspond to those use in Fig 5.

| **Participant** | **Convergent clonotypes** | **# of PBs in clonotype** | **Convergent group** | **Expressed public mAb** |
| --- | --- | --- | --- | --- |
| D#008 | IGHV3-7/D4-17/J6/IGLV3-21/J1 | 8 | pubCDR3-4 | #7665 |
|  | IGHV3-7/D4-17/J6/IGLV3-21/J3 | 1 | pubCDR3-4 |  |
| D#015 | n/a | n/a | n/a |  |
| D#030 | IGHV3-9/D2-15/J6/IGLV1-40/J3 | 8 | pubCDR3-2 | #3978 |
|  | IGHV3-9/D2-21/J6/IGLV1-40/J3 | 1 | pubCDR3-2 |  |
|  | IGHV3-9/D2-15/J6/IGLV1-40/J2 | 1 | pubCDR3-2 |  |
| D#038 | n/a | n/a | n/a |  |
| D#070 | n/a | n/a | n/a |  |
| D#082 | n/a | n/a | n/a |  |
| D#085 | IGHV3-23/D3-3/J6/IGLV1-44/J1 | 4 | pubCDR3-1 |  |
|  | IGHV3-23/D3-3/J6/IGLV1-51/J2 | 2 | pubCDR3-1 |  |
|  | IGHV3-23/D3-3/J6/IGLV1-51/J3 | 2 | pubCDR3-1 |  |
| D#089 | IGHV3-23/D3-3/J6/IGLV1-51/J2 | 8 | pubCDR3-1 |  |
|  | IGHV3-23/D3-3/J6/IGLV2-14/J2 | 6 | pubCDR3-1 |  |
|  | IGHV3-23/D3-3/J6/IGLV1-40/J3 | 1 | pubCDR3-1 |  |
|  | IGHV3-23/D3-3/J6/IGLV1-44/J2 | 1 | pubCDR3-1 |  |
| D#099 | IGHV1-2/D3-22/J4/IGLV3-1/J3 | 2 | pubCDR3-3 |  |
| D#102 | IGHV3-9/D2-15/J6/IGLV1-40/J2 | 6 | pubCDR3-2 |  |
|  | IGHV1-2/D3-22/J4/IGLV3-1/J3 | 1 | pubCDR3-3 |  |
| D#103 | IGHV3-23/D3-3/J6/IGLV1-44/J2 | 2 | pubCDR3-1 |  |
|  | IGHV3-23/D3-3/J6/IGLV1-51/J1 | 1 | pubCDR3-1 |  |
|  | IGHV3-23/D3-3/J6/IGLV2-14/J2 | 1 | pubCDR3-1 |  |
|  | IGHV3-9/D2-2/J6/IGLV1-40/J2 | 1 | pubCDR3-2 |  |
| D#108 | n/a | n/a | n/a |  |
| D#113 | IGHV1-2/D3-22/J4/IGLV3-1/J2 | 3 | pubCDR3-3 |  |
|  | IGHV3-7/D4-17/J6/IGLV3-21/J1 | 38 | pubCDR3-4 | #5589 |
| D#118 | n/a | n/a | n/a |  |
| D#120 | n/a | n/a | n/a |  |
| D#122 | IGHV3-9/D2-15/J6/IGLV1-40/J3 | 1 | pubCDR3-2 |  |
| D#127 | IGHV1-2/D3-22/J4/IGLV3-1/J1 | 20 | pubCDR3-3 |  |
|  | IGHV1-2/D3-22/J4/IGLV3-1/J3 | 1 | pubCDR3-3 |  |
|  | IGHV1-2/D3-22/J4/IGLV3-1/J2 | 1 | pubCDR3-3 |  |
