# Supplementary material for: Convergent antibody evolution and clonotype expansion following influenza virus vaccination
Source: PLoS One. 2021 Feb 22;16(2):e0247253. doi: 10.1371/journal.pone.0247253 (PMC7899375; doi:10.1371/journal.pone.0247253)
Supplement: S4 Table — (DOCX) [file pone.0247253.s013.docx]

**S4 Table.** **Key resources.**

| ***REAGENT or RESOURCE*** | ***SOURCE*** | ***IDENTIFIER*** |
| --- | --- | --- |
| **Antibodies** | | |
| IgD BV421 | BioLegend | Cat# 348226 |
| CD20 BV650 | BioLegend | Cat# 302334 |
| CD3 FITC | BioLegend | Cat# 300306 |
| CD14 FITC | BioLegend | Cat# 301804 |
| CD38 PE-Dazzel | BioLegend | Cat# 303538 |
| CD27 PE-Cy7 | BioLegend | Cat# 124216 |
| CD19 APC-Fire | BioLegend | Cat# 302258 |
|  |  |  |
| **Bacterial and Virus Strains** | | |
| IAV-H1N1 (A/South Carolina/1918) | Laboratory of Ted Ross, University of Georgia | Lot BCL-112915SC1918VLP |
| IAV-H1N1 (A/Weiss/1/1943) | VIRAPUR | Lot VP-H1517B |
| IAV-H1N1 (A/Fort Monmouth/1/1947) | VIRAPUR | Lot VP-I1528A |
| IAV-H1N1 (A/Denver/1/1957) | VIRAPUR | Lot VP-H1517A |
| IAV-H1N1 (A/New Jersey/8/1976) | VIRAPUR | Lot VP-G1509B |
| IAV-H1N1 PR8 (A/USSR/90/1977) | VIRAPUR | Lot VP-G1509C |
| IAV-H1N1 PR8 (A/Brazil/11/1978) | VIRAPUR | Lot VP-G1501A |
| IAV-H1N1 PR8 (A/Chile/1/1983) | VIRAPUR | Lot VP-J1509A |
| IAV-H1N1 (A/Singapore/6/1986) | VIRAPUR | Lot VP-L1509C |
| IAV-H1N1 (A/Texas/39/1991) | VIRAPUR | Lot VP-I1530C |
| IAV-H1N1 PR8 (A/Beijing/262/1995) | VIRAPUR | Lot VP-H1517C |
| IAV-H1N1 PR8 (A/New Caledonia/20/1999) | VIRAPUR | Lot VP-G1509A |
| IAV-H1N1 PR8 (A/Solomon Islands/3/2006) | VIRAPUR | Lot VP-F1522A |
| IAV-H1N1 PR8 (A/Brisbane/59/2007) | VIRAPUR | Lot VP-F1524A |
| IAV-H1N1 PR8 (A/California/07/2009) | VIRAPUR | Lot VP-F1512B |
| IAV-H1N1 (A/Michigan/45/2015) | VIRAPUR | Lot VP-K1610A |
| IAV-H3N2 (A/Hong Kong/8/1968) | VIRAPUR | Lot VP-L1503A |
| IAV-H3N2 (A/Port Chalmers/1/1973) | VIRAPUR | Lot VP-K1516D |
| IAV-H3N2 PR8 (A/Texas/1/1977) | Laboratory of Ted Ross, University of Georgia | Lot AB-2315TX77MDCK1 |
| IAV-H3N2 PR8 (A/Mississippi/1/1985) | Laboratory of Ted Ross, University of Georgia | Lot BCL-12315M85MDCK1 |
| IAV-H3N2 (A/Sichuan/60/1989) | Laboratory of Ted Ross, University of Georgia | BCL-012615SCH89EP1 |
| IAV-H3N2 (A/Shangdong/9/1993) | Laboratory of Ted Ross, University of Georgia | Lot BCL-2615S93MDCK1 |
| IAV-H3N2 PR8 (A/Nanchang/933/1995) | VIRAPUR | Lot VP-K1622A |
| IAV-H3N2 (A/Sydney/5/1997) | Laboratory of Ted Ross, University of Georgia | Lot BCL-020116SY97EP1 |
| IAV-H3N2 (A/Panama/2007/1999) | VIRAPUR | Lot VP-L1503B |
| IAV-H3N2 (A/New York/55/2004) | VIRAPUR | Lot VP-C1729E |
| IAV-H3N2 (A/Wisconsin/67/2005) | VIRAPUR | Lot VP-J1519C |
| IAV-H3N2 PR8 (A/Uruguay/716/2007) | VIRAPUR | Lot VP-I1606C |
| IAV-H3N2 (A/Perth/16/2009) | VIRAPUR | Lot VP-H1507A |
| IAV-H3N2 (A/Victoria/361/2011) | VIRAPUR | Lot VP-J1519B |
| IAV-H3N2 (A/Texas/50/2012) | VIRAPUR | Lot VP-I1508B |
| IAV-H3N2 (A/Switzerland/9715293/2013) | VIRAPUR | Lot VP-J1506B |
| IAV-H3N2 PR8 (A/Hong Kong/4801/2014) | VIRAPUR | Lot VP-E1603A |
| IBV-Yam (B/Yamagata/16/1988) | Laboratory of Ted Ross, University of Georgia | Lot EE-YM88-030618 |
| IBV-Yam (B/Harbin/7/1994) | Laboratory of Ted Ross, University of Georgia | Lot EE-HB94-030618 |
| IBV-Yam (B/Sichuan/379/1999) | Laboratory of Ted Ross, University of Georgia | Lot EE-SIC99.2-080718 |
| IBV-Yam (B/Florida/4/2006) | Laboratory of Ted Ross, University of Georgia | Lot EE-FL06-022018 |
| IBV-Yam (B/Wisconsin/1/2010) | Laboratory of Ted Ross, University of Georgia | Lot EE-WI10-043018 |
| IBV-Yam (B/Texas/6/2011) | Laboratory of Ted Ross, University of Georgia | Lot EE-TX11-122017 |
| IBV-Yam (B/Massachusetts/02/2012) | Laboratory of Ted Ross, University of Georgia | Lot EE-MA12-112117 |
| IBV-Yam (B/Phuket/3073/2013) | Laboratory of Ted Ross, University of Georgia | Lot EE-PH13-112117 |
| IBV-Vic (B/Victoria/2/1987) | Laboratory of Ted Ross, University of Georgia | Lot EE-VIC87-070319 |
| IBV-Vic (B/Hong Kong/330/2001) | Laboratory of Ted Ross, University of Georgia | Lot EE-HK01-022018 |
| IBV-Vic (B/Malaysia/2506/2004) | Laboratory of Ted Ross, University of Georgia | Lot EE-MY04-122017 |
| IBV-Vic (B/Victoria/304/2006) | Laboratory of Ted Ross, University of Georgia | Lot EE-VIC06-112018 |
| IBV-Vic (B/Brisbane/60/2008) | Laboratory of Ted Ross, University of Georgia | Lot EE-BR08-031418 |
| IBV-Vic (B/Colorado/06/2017) | Laboratory of Ted Ross, University of Georgia | Lot EE-CO17-052318 |
|  |  |  |
| **Biological Samples** |  |  |
| Human PBMCs |  | Reviewed and approved by the IRB of the University of Georgia |
|  |  |  |
| **Chemicals, Peptides, and Recombinant Proteins** | | |
| H1N1 A/Chile/1/1983 (Chile/83) rHA | Laboratory of Ted Ross, University of Georgia | [22,23] |
| H1N1 A/Singapore/6/1986 (Sing/86) rHA | Laboratory of Ted Ross, University of Georgia | [22,23] |
| A/Texas/36/1991 (TX/91) rHA | Florian Krammer’s laboratory stock | [62] |
| H1N1 A/New Caledonia/20/1999 (NC/99) rHA | Laboratory of Ted Ross, University of Georgia | [22,23] |
| H1N1 A/Solomon Islands/3/2006 (SI/06) rHA | BEI RESOURCES | NR-15170 |
| H1N1 A/Brisbane/59/2007 (Brisb/07) rHA | Laboratory of Ted Ross, University of Georgia | [22,23] |
| H1N1 A/California/07/2009 (CA/09) rHA | Laboratory of Ted Ross, University of Georgia | [20] |
| H1N1 A/Michigan/45/2015 (MI/15) rHA | Laboratory of Ted Ross, University of Georgia | [22,23] |
| H1N1 A/Brisbane/02/2018 (Brisb/18) rHA | Laboratory of Ted Ross, University of Georgia | N/A |
| Chimeric rHA with HA1 from H6N1 A/Mallard/Sweden/81/2002 and HA2 from CA/09 (cH6/1) | Laboratory of Ted Ross, University of Georgia | [20,22,23] |
| H3N2 A/Panama/07/1999 (Pan/99) rHA | Laboratory of Ted Ross, University of Georgia | N/A |
| H3N2 A/Wyoming/03/2003 (WY/03) rHA | Protein Sciences | Lot n. 868-078 |
| H3N2 A/New York/55/2004 (NY/04) rHA | Protein Sciences | Lot n. 1099-044 |
| H3N2 A/Wisconsin/67/2005 (WI/05) rHA | Protein Sciences | Lot n. 1099-087 |
| H3N2 A/Uruguay/716/2007 (Uru/07) rHA | Protein Sciences | Lot n. 1099-025 |
| H3N2 A/Brisbane/10/2007 (Brisb/07) rHA | Protein Sciences | Lot n. 45-10008 |
| H3N2 A/Perth/16/2009 (Perth/09) rHA | Protein Sciences | Lot n. 1294-043 |
| H3N2 A/Victoria/361/2011 (Vic/11) rHA | Laboratory of Ted Ross, University of Georgia | N/A |
| H3N2 A/Texas/50/2012 (TX/12) rHA | Laboratory of Ted Ross, University of Georgia | N/A |
| H3N2 A/Switzerland/9715293/2013 (Switz/13) rHA | Laboratory of Ted Ross, University of Georgia | N/A |
| H3N2 A/Hong Kong/4801/2014 (HK/14) rHA | Protein Sciences | Lot n. 1099-112 |
| H3N2 A/Singapore/ANFIMH-16-0019/2016 (Sing/16) rHA | Laboratory of Ted Ross, University of Georgia | N/A |
| H3N2 A/Kansas/14/2017 (KS/17) rHA | Laboratory of Ted Ross, University of Georgia | N/A |
| H3N2 A/Switzerland/8060/2017 (Switz/17) rHA | Laboratory of Ted Ross, University of Georgia | N/A |
| H3N2 A/South Australia/34/2019 (S.Aus/19) rHA | Laboratory of Ted Ross, University of Georgia | N/A |
| cH5/3 (chimeric rHA with HA1 from H5N1 A/Vietnam/1203/2004 and HA2 from Perth/09) | Florian Krammer’s laboratory stock | [76] |
| cH7/3 (chimeric rHA with HA1 from H7N9 A/Anhui/1-YK/2013 and HA2 from Perth/09) | Laboratory of Ted Ross, University of Georgia | [20] |
| B-Yam B/Jilin/20/2003 (Jilin/03) rHA | BEI RESOURCES | NR-19242 |
| B-Yam B/Florida/04/2006 (FL/06) rHA | Protein Sciences | Lot n. 45-08020 |
| B-Yam B/Wisconsin/01/2010 (WI/10) rHA | Protein Sciences | Lot n. 1266-030 |
| B-Yam B/Massachusetts/02/2012 (MA/12) rHA | Protein Sciences | Lot n. 1208-106 |
| B-Yam B/Phuket/3073/2013 (Phu/13) rHA | Laboratory of Ted Ross, University of Georgia | [20] |
| B-Vic B/Malaysia/2506/2004 (Mal/04) rHA | Protein Sciences | Lot n. 45-11006 |
| B-Vic B/Ohio/01/2005 (OH/05) rHA | BEI RESOURCES | NR-19243 |
| B-Vic B/Brisbane/60/2008 (Brisb/08) rHA | Protein Sciences | Lot n. 45-11027 |

**S1 References cited only in the Supplemental information.**

76. Margine I, Hai R, Albrecht RA, Obermoser G, Harrod AC, Banchereau J, et al. H3N2 influenza virus infection induces broadly reactive hemagglutinin stalk antibodies in humans and mice. J Virol. 2013;87: 4728-4737.
