# Supplementary material for: Convergent antibody evolution and clonotype expansion following influenza virus vaccination
Source: PLoS One. 2021 Feb 22;16(2):e0247253. doi: 10.1371/journal.pone.0247253 (PMC7899375; doi:10.1371/journal.pone.0247253)
Supplement: S1 Text — (DOCX) [file pone.0247253.s014.docx]

**S1 Text.** **Strain abbreviations.**

The following influenza virus strain abbreviations are used:

H1N1, A/Chile/1/1983 (Chile/83); A/Singapore/6/1986 (Sing/86); A/Texas/36/1991 (TX/91); A/New Caledonia/20/1999 (NC/99); A/Solomon Islands/3/2006 (SI/06); A/Brisbane/59/2007 (Brisb/07); A/California/07/2009 (CA/09); A/Michigan/45/2015 (MI/15); A/Brisbane/02/2018 (Brisb/18); chimeric HA with HA1 from H6N1 A/Mallard/Sweden/81/2002 and HA2 from CA/09 (cH6/1); H3N2, A/Panama/07/1999 (Pan/99); A/Wyoming/03/2003 (WY/03); A/New York/55/2004; A/Wisconsin/67/2005 (WI/05); A/Uruguay/716/2007; A/Brisbane/10/2007 (Brisb/07); A/Perth/16/2009 (Perth/09); A/Victoria/361/2011 (Vic/11); A/Texas/50/2012 (TX/12); A/Switzerland/9715293/2013 (Switz/13); A/Singapore/ANFIMH-16-0019/2016 (Sing/16); A/Hong Kong/4801/2014 (HK/14); A/Kansas/14/2017 (KS/17); A/Switzerland/8060/2017 (Switz/17); A/South Australia/34/2019 (S.Aus/19); chimeric HA with HA1 from H5N1 A/Vietnam/1203/2004 or H7N9 A/Anhui/1-YK/2013 for cH5/3 and cH7/3, respectively, and HA2 from Perth/09; IBV, B/Jilin/20/2003 (Jilin/03); B/Florida/04/2006 (FL/06); B/Wisconsin/01/2010 (WI/10); B/Massachusetts/02/2012 (MA/12); B/Phuket/3073/2013; B/Malaysia/2506/2004 (Mal/04); B/Ohio/01/2005 (OH/05); B/Brisbane/60/2008 (Brisb/08).
