# Supplementary material for: Convergent antibody evolution and clonotype expansion following influenza virus vaccination
Source: PLoS One. 2021 Feb 22;16(2):e0247253. doi: 10.1371/journal.pone.0247253 (PMC7899375; doi:10.1371/journal.pone.0247253)
Supplement: Attachment — Submitted filename: Forgacs.PLoS One Response to reviewers.docx [file pone.0247253.s015.docx]

Athens, GA - January 29, 2021

Dear Editor:

We are resubmitting a revised version of our manuscript ID PONE-D-20-32620 entitled ***“*Convergent antibody evolution and clonotype expansion following influenza virus vaccination*”*** to PLoS ONE after having addressed the revisions that were suggested by you and the reviewers. Below, please find a point-by-point reply to the referee comments.

**Editor comments:**

1. We would like to update the Financial Disclosure section to state the following:

This study was supported by the NIH contract HHSN272201400004C (NIAID Centers of Excellence for Influenza Research and Surveillance, CEIRS). The content is solely the responsibility of the authors and does not necessarily represent the official views of the NIH. The funder provided support in the form of salaries for authors [DF, RBA, GAS, GAK], but did not have any additional role in the study design, data collection and analysis, decision to publish, or preparation of the manuscript. The specific roles of these authors are articulated in the ‘Author contributions’ section. Some of the authors are affiliated with Atreca, Inc., however, they provided no funding for the study, only assisted with the data collection and the preparation of the manuscript.

(Please note also that in the previous submission, GACRC was erroneously included in the Financial Disclosure section. While they provided assistance, it was all technical and not financial, so the statement belongs in the Acknowledgements but not in this section.)

2. In accordance to your request, we would also like to update the Competing Interests Statements to report the following:

Some of the authors are affiliated with Atreca, Inc., but the company provided no funding for the study and no competing interests exist. The commercial affiliation of those authors does not alter our adherence to PLoS ONE policies on sharing data and materials.

3. The statements including the phrase “data not shown” have been replaced by the appropriate references including the data or relating to the statement (lines 534 and 631).

**Reviewer comments:** 

Reviewer #1 (Response to Questions):

To the question “*Have the authors made all data underlying the findings in their manuscript fully available?*”, Reviewer #1 answered no. This was correct at the time of the review as only placeholders were left in the manuscript while NCBI authenticated and released the 15,554 novel nucleotide sequences provided by our study. They are now all publicly available in GenBank as a Target Locus Study under accession numbers KEOV00000000 and KEOU00000000. References to those accession numbers have been included in the manuscript (line 190).

Reviewer #1 (Comments to the Author):

1. *Please show error bars for the HAI data in Figure 1B*

We thank the reviewer for having noticed that and we apologize for our oversight. Error bars have now been added to the HAI panel in Figure 1B, along with the legend now stating that all samples were run in triplicate (line 265).

2. *Please provide specific percentages for IgG2 and IgG3*.

Specific percentages have been provided for IgG2 and IgG3 in lines 271-272.

Reviewer #2 (Comments to the Author):

1. *Public clonotypes were defined as ...."PBs whose heavy chain variable region nucleotide pairwise identity was over 80%, and HCDR3 peptide sequence pairwise identity was over 75% compared to at least one other PB from a different participant in the same group". This is very permissive. What is the justification for such a low threshold for HCDR3 aa identity? Did the clonotypes considered as "public" share the same VJ genes. Analysis of public clonotypes with more stringent criteria, ie same V same J and >85% aa identity needs to be performed and reported.*

Public convergent clonotypes are defined by three criteria, all of which had to be true in order to be considered part of the same public clonotype:

1. As Reviewer #2 stated, all B cell belonging to the same clonotype must have had identical heavy chain V and J genes. The text has been appended to include that distinction.
2. The minimum heavy chain nucleotide percent identities between two B cell had to be a minimum of 80%.
3. And in addition to strengthen our criteria, the HCDR3 amino acid identity between donors was set at a minimum of 75%.

These criteria are consistent with what is recently published in the literature (e.g. Galson *et al.*, 2020. *Front. Imm*.; Setliff *et al.*, 2018. *Cell Host Microbe*). Increasing this cutoff to the recommended 85% would only exclude two of the 98 B cells that we found to be convergent: one from pubCDR3-1 that otherwise has an 83% heavy chain nucleotide identity, the other from pubCDR3-4 which has a staggering 93% heavy chain nucleotide identity. As both of these B cells have the same V and J genes as other members of their convergent clonotypes, as well as heavy chain nucleotide identities exceeding the published norm, we still feel strongly about including them in the analysis. However, we have included a clear description and rationale in the appropriate section (lines 230-234).

2. *Given that public VH were paired with different VL was there any analysis of Mabs to show that the binding specificity was primarily VH driven? Not an essential point, but at least should be discussed?*

We agree that this point should be touched on, and though we have not analyzed VH/VL specificity for the purposes of this manuscript, we included a paragraph to address this point (lines 578-587).

3. *The text is excessively long, especially the Discussion. The text should be reduced by at least 30%.*

We have done our best to shorten the Introduction, Materials and methods, as well as the Discussion sections. Though we understand that the manuscript is long, we do believe it to be free of superfluous details. We truly appreciate the lack of strict word limits for PLoS ONE publications.

Sincerely,

Ted M. Ross, PhD

GRA Eminent Scholar in Infectious Diseases

Director - Center for Vaccines and Immunology

Professor - Department of Infectious Diseases

University of Georgia
